# Supplementary figures and images for: Lysosome damage triggers direct ATG8 conjugation and ATG2 engagement via non-canonical autophagy
Source: J Cell Biol. 2023 Oct 5;222(12):e202303078. doi: 10.1083/jcb.202303078 (PMC10561555; doi:10.1083/jcb.202303078)

Source Data Figure 1

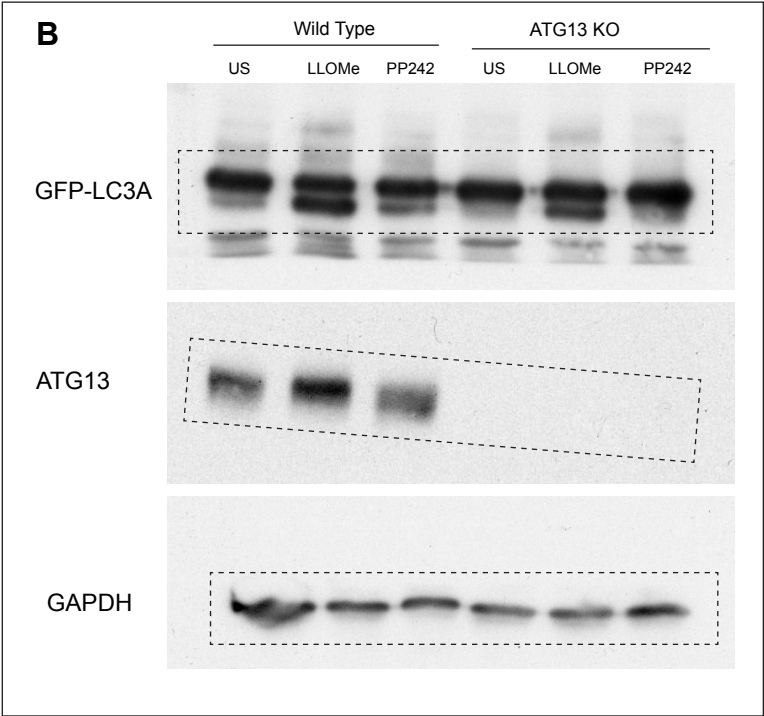

Supplement: SourceData F1 — is the source file for Fig. 1. [file JCB_202303078_SourceDataF1.pdf]

Source Data Figure 2

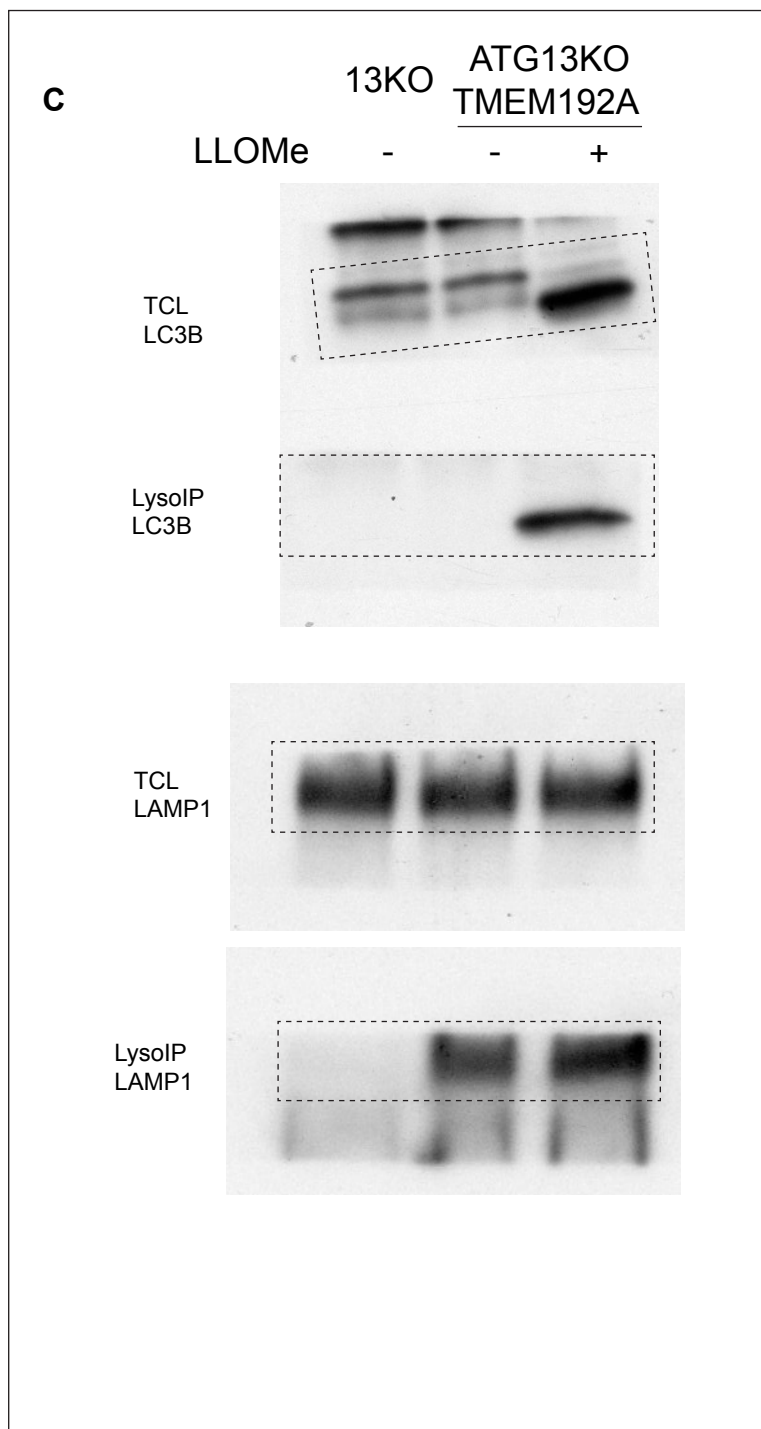

Supplement: SourceData F2 — is the source file for Fig. 2. [file JCB_202303078_SourceDataF2.pdf]

Source Data Figure 3

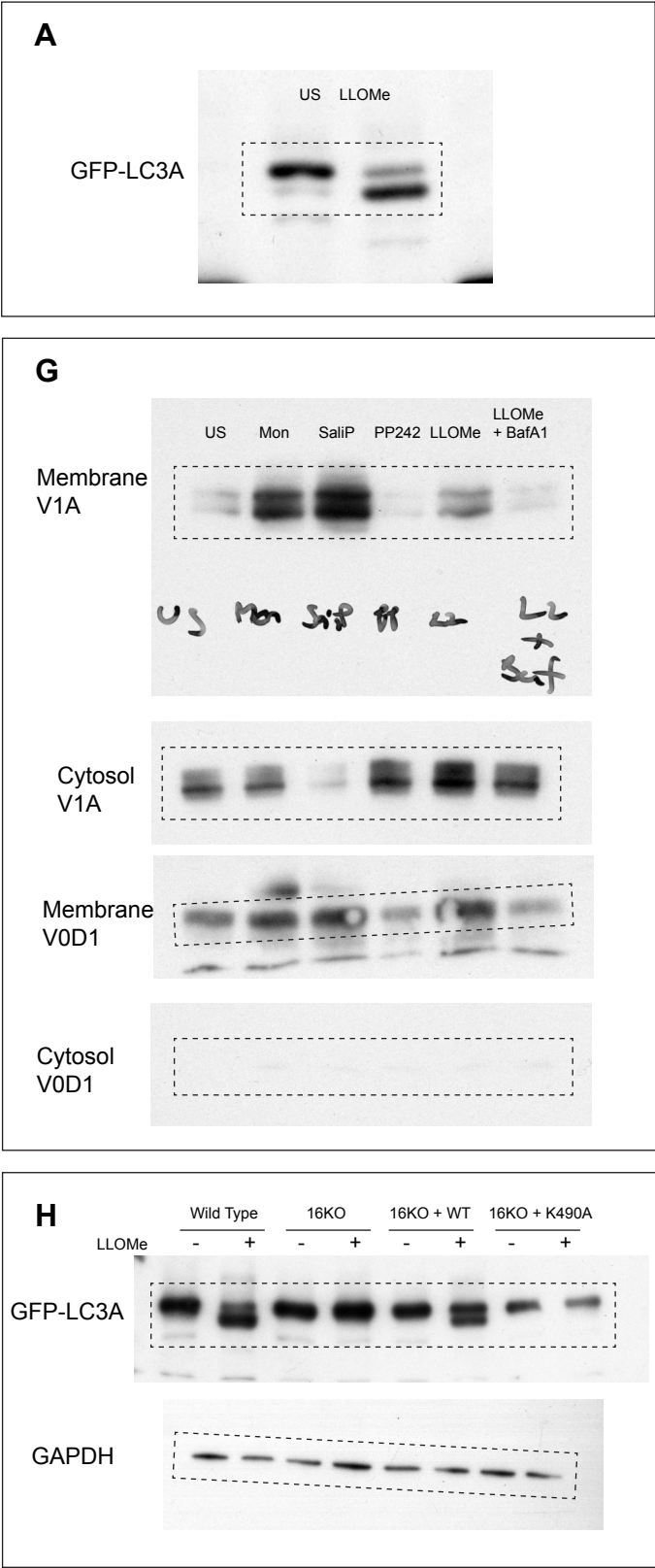

Supplement: SourceData F3 — is the source file for Fig. 3. [file JCB_202303078_SourceDataF3.pdf]

Source Data Figure 4

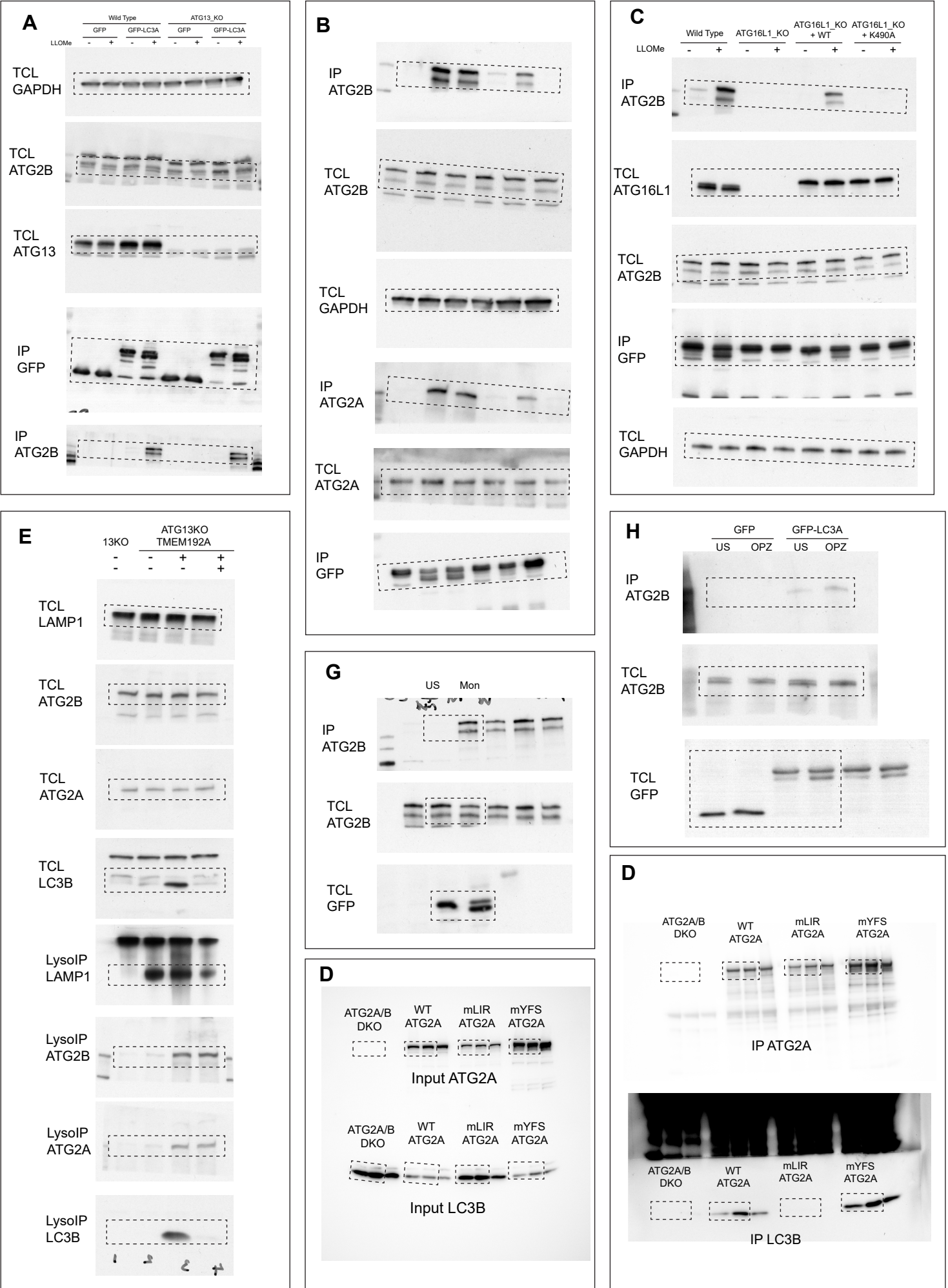

Supplement: SourceData F4 — is the source file for Fig. 4. [file JCB_202303078_SourceDataF4.pdf]

Source Data Figure S2

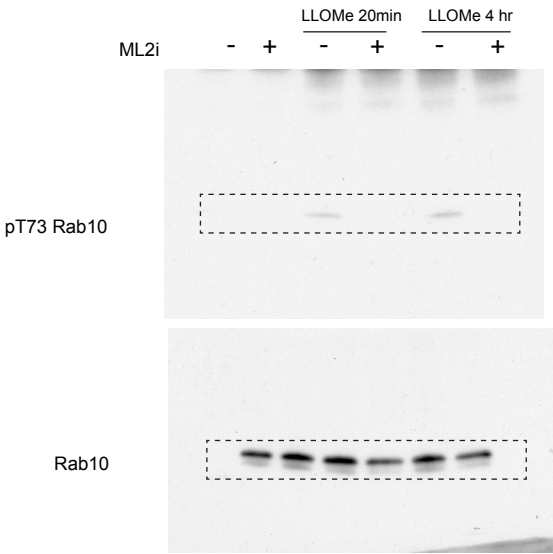

Supplement: SourceData FS2 — is the source file for Fig. S2. [file JCB_202303078_SourceDataFS2.pdf]
